# Uncovering the missing links: autoimmunity and infection in pediatric arterial ischemic stroke

**DOI:** 10.1055/s-0045-1815740

**Published:** 2026-04-15

**Authors:** Christian Messina

**Affiliations:** 1 Azienda Sanitaria Provinciale Catania, Catania, Italy.

Dear Editor,

We read with great interest the article by Bostancı et al., 1 who retrospectively analyzed 23 pediatric cases of arterial ischemic stroke (AIS) admitted to a tertiary hospital over a 4-year period. The study 1 highlights the diverse clinical presentations of AIS in children, ranging from neurological deficits and seizures to less typical manifestations such as hiccups, anisocoria, and gaze abnormalities. The radiological evaluation revealed that cerebral infarctions were most frequently located in the cerebrum, while genetic predispositions such as *methylenetetrahydrofolate reductase* ( *MTHFR* ) mutations and comorbid cardiac anomalies were also reported. 1 The authors 1 further emphasize the value of cranial computed tomography (CT) scanning, particularly in settings where magnetic resonance imaging (MRI) is not readily accessible for pediatric populations. While the findings are clinically relevant and contribute to raising awareness of early recognition and management, several limitations and clarifications need to be addressed in order to strengthen the interpretation of the results.

In their study, the authors 1 collected a series of laboratory and instrumental data, including blood count, levels of C-reactive protein (CRP), serum electrolytes, triglycerides, total cholesterol, prothrombin time, activated partial thromboplastin time, levels of protein C, protein S, and homocysteine, electrocardiography, echocardiography, brain CT, and MRI. However, several important parameters that could have substantially contributed to clarifying the underlying etiology of pediatric AIS were not assessed. Most notably, autoimmune markers were not included, such as antiphospholipid antibodies, antinuclear antibodies (ANAs), extractable nuclear antigen (ENA) screening, perinuclear antineutrophil cytoplasmic antibodies (p-ANCAs), cytoplasmic ANCAs (c-ANCAs), and lupus anticoagulant. These antibodies are well-recognized risk factors for ischemic stroke and, importantly, for recurrent ischemic stroke, with their coexistence markedly amplifying the thrombotic risk. 2 Furthermore, the measurement of the erythrocyte sedimentation rate (ESR) would also have been of value, as these immunological and inflammatory markers are frequently associated with systemic vasculitides. 2 3 Notably, pediatric vasculitides—including granulomatosis with polyangiitis (formerly *Wegener's granulomatosis* ), microscopic polyangiitis, and eosinophilic granulomatosis with polyangiitis (previously *Churg-Strauss syndrome* )—may involve the central nervous system, with ischemic manifestations in 10 to 20% of the cases. 3 Therefore, the inclusion of such parameters would have provided a more comprehensive assessment of potential autoimmune and inflammatory contributors to AIS in children.

Another important factor to consider is the potential role of infections. The ages of the study cohort ranged from 1 month to 18 years, which represents a critical period for the onset of infectious diseases, particularly exanthematous illnesses, due to increased exposure to the external environment and to early social interactions. Thus, it would have been highly valuable to investigate infectious causes, especially viral infections. Among these, members of the herpesvirus family are particularly relevant. Moreover, given that the data were collected between 2016 and 2020, part of the study period coincided with the outbreak of the severe acute respiratory syndrome coronavirus 2 (SARS-CoV-2) pandemic. Herpesviruses and SARS-CoV-2 have been associated with an increased risk of ischemic stroke. A well-documented example is the varicella-zoster virus (VZV), which significantly elevates the risk of stroke in the postinfectious period. 4 Therefore, evaluating patients' infectious history, together with dermatological manifestations that may serve as clinical clues, could have provided further meaningful insights into the potential etiological mechanisms of AIS in this pediatric population.

In conclusion, while the work by Bostancı et al. 1 provides valuable data on pediatric AIS, its scope remains limited by the absence of key immunological and infectious evaluations. Autoantibodies, markers of systemic inflammation, and viral triggers represent critical variables that may substantially alter risk stratification and recurrence patterns. Future studies incorporating these parameters, alongside comprehensive clinical and laboratory assessments, will be essential to fully elucidate the multifactorial etiology of pediatric stroke and to design more effective preventive and therapeutic strategies.
